# Supplementary material for: Energy-efficient pathway for selectively exciting solute molecules to high vibrational states via solvent vibration-polariton pumping
Source: Nat Commun. 2022 Jul 20;13:4203. doi: 10.1038/s41467-022-31703-8 (PMC9300737; doi:10.1038/s41467-022-31703-8)
Supplement: Supplementary file 1 — Supplementary Information [file 41467_2022_31703_MOESM1_ESM.pdf]

Supplementary Information

"Energy-Efficient Pathway for Selectively Exciting Solute Molecules to High Vibrational States via Solvent Vibration-Polariton Pumping"

Tao E. Li,[1,2,*] Abraham Nitzan,[1,3,*] and Joseph E. Subotnik[1,*]

[1]*Department of Chemistry, University of Pennsylvania, Philadelphia, Pennsylvania 19104, USA*

[2]*Department of Chemistry, Yale University, New Haven, Connecticut, 06520, USA*

[3]*School of Chemistry, Tel Aviv University, Tel Aviv 69978, Israel*

SUPPLEMENTARY METHODS

Following [1, 2], given a full-quantum photon-electron-nuclei Hamiltonian in the dipole gauge and under long-wave approximation, cavity molecular dynamics (CavMD) simulations invoke the Born–Oppenheimer approximation. The full quantum Hamiltonian is projected onto the electronic ground state, leading to:

$$\hat{H}_{\mathrm{QED}}^{\mathrm{G}} = \hat{H}_{\mathrm{M}}^{\mathrm{G}} + \hat{H}_{\mathrm{F}}^{\mathrm{G}}. \tag{S1a}$$

Here, $\hat{H}_{\mathrm{M}}^{\mathrm{G}}$ denotes the standard ground-state molecular Hamiltonian, which is composed of kinetic energies of nuclei ($\hat{\mathbf{P}}_{nj}^2/2M_{nj}$), intramolecular interactions ($\hat{V}_g^{(n)}$) and pairwise intermolecular interactions ($\hat{V}_{\mathrm{inter}}^{(nl)}$):

$$\hat{H}_{\mathrm{M}}^{\mathrm{G}} = \sum_{n=1}^{N}\left(\sum_{j\in n}\frac{\hat{\mathbf{P}}_{nj}^2}{2M_{nj}} + \hat{V}_g^{(n)}(\{\hat{\mathbf{R}}_{nj}\})\right) + \sum_{n=1}^{N}\sum_{l>n}\hat{V}_{\mathrm{inter}}^{(nl)}, \tag{S1b}$$

where $\hat{\mathbf{P}}_{nj}$, $\hat{\mathbf{R}}_{nj}$, and M_{nj} denote the momentum operator, position operator, and mass for the j-th nucleus in molecule n. $\hat{H}_{\mathrm{F}}^{\mathrm{G}}$ denotes the field-related Hamiltonian [1, 2] where each cavity photon mode with wave vector $\mathbf{k}$ and polarization direction $\boldsymbol{\xi}_\lambda$ interacts with the total dipole moment of the molecular subsystem:

$$\hat{H}_{\mathrm{F}}^{\mathrm{G}} = \sum_{k,\lambda}\frac{\hat{\tilde{p}}_{k,\lambda}^2}{2m_{k,\lambda}} + \frac{1}{2}m_{k,\lambda}\omega_{k,\lambda}^2\left(\hat{\tilde{q}}_{k,\lambda} + \sum_{n=1}^{N}\frac{\hat{d}_{ng,\lambda}}{\omega_{k,\lambda}\sqrt{\Omega\epsilon_0 m_{k,\lambda}}}\right)^2. \tag{S1c}$$

Here, $\hat{\tilde{p}}_{k,\lambda}$, $\hat{\tilde{q}}_{k,\lambda}$, $\omega_{k,\lambda}$, and $m_{k,\lambda}$ denote the momentum operator, position operator, frequency, and the auxiliary mass for each cavity photon. Ω denotes the volume for the cavity, ϵ_0 denotes the vacuum permittivity, and $\hat{d}_{ng,\lambda}$ denotes the electronic ground-state dipole operator for molecule n ($\hat{\mathbf{d}}_n$) projected along the direction of $\boldsymbol{\xi}_\lambda$: $\hat{d}_{ng,\lambda} \equiv \hat{\mathbf{d}}_n \cdot \boldsymbol{\xi}_\lambda$. Compared with usual light-matter Hamiltonian [3–6] where there is no auxiliary mass $m_{k,\lambda}$ term for the photonic operators ($\hat{p}_{k,\lambda}$ and $\hat{q}_{k,\lambda}$), in Eq. (S1c) $m_{k,\lambda}$ is introduced because standard molecular dynamics packages require this quantity. One can obtain $m_{k,\lambda}$ by simply rescaling the conventional photonic operators as follows: $\hat{q}_{k,\lambda} \equiv \sqrt{m_{k,\lambda}}\hat{\tilde{q}}_{k,\lambda}$ and $\hat{p}_{k,\lambda} \equiv \hat{\tilde{p}}_{k,\lambda}/\sqrt{m_{k,\lambda}}$. Hence, the use of the auxiliary mass does not alter any dynamics. In simulations, we take $m_{k,\lambda} = 1$ a.u. (atomic units) for simplicity.

* taoli@sas.upenn.edu

* anitzan@sas.upenn.edu

* subotnik@sas.upenn.edu

In Eq. (S1c), we can further define the coupling strength between each cavity photon and each individual molecule as

$$\varepsilon_{k,\lambda} \equiv \sqrt{m_{k,\lambda}\omega_{k,\lambda}^2/\Omega\epsilon_0}. \tag{S2}$$

In order to efficiently simulate the above quantum Hamiltonian in Eq. (S1), we reduce all of the operators to classical observables and obtain the equations of motion as follows

$$M_{nj}\ddot{\mathbf{R}}_{nj} = \mathbf{F}_{nj}^{(0)} - \sum_{k,\lambda}\left(\varepsilon_{k,\lambda}\widetilde{q}_{k,\lambda} + \frac{\varepsilon_{k,\lambda}^2}{m_{k,\lambda}\omega_{k,\lambda}^2}\sum_{l=1}^{N} d_{lg,\lambda}\right)\frac{\partial d_{ng,\lambda}}{\partial \mathbf{R}_{nj}}, \tag{S3a}$$

$$m_{k,\lambda}\ddot{\widetilde{q}}_{k,\lambda} = -m_{k,\lambda}\omega_{k,\lambda}^2\widetilde{q}_{k,\lambda} - \varepsilon_{k,\lambda}\sum_{n=1}^{N} d_{ng,\lambda}. \tag{S3b}$$

Here, $\mathbf{F}_{nj}^{(0)} = -\partial V_g^{(n)}/\partial \mathbf{R}_{nj} - \sum_{l\neq n}\partial V_{\text{inter}}^{(nl)}/\partial \mathbf{R}_{nj}$ denotes the molecular part of the force on each nuclei, i.e., the nuclear force outside the cavity.

a. Periodic boundary conditions VSC inside a Fabry–Pérot microcavity usually involves a macroscopic number (say, $10^9 \sim 10^{11}$) of molecules. For such a large molecular system, it is very expensive if we propagate (S3) directly. Hence, we further apply periodic boundary conditions, i.e., we assume that the molecular subsystem can be divided into N_{cell} identical periodic cells. In detail, for Eq. (S3) we approximate the total dipole moment of molecules as $\sum_{n=1}^{N} d_{ng,\lambda} = N_{\text{cell}}\sum_{n=1}^{N_{\text{sub}}} d_{ng,\lambda}$, where $N_{\text{sub}} = N/N_{\text{cell}}$ denotes the number of molecules in a single cell. By further denoting $\widetilde{\widetilde{q}}_{k,\lambda} = \widetilde{q}_{k,\lambda}/\sqrt{N_{\text{cell}}}$, $\widetilde{\varepsilon}_{k,\lambda} = \sqrt{N_{\text{cell}}}\varepsilon_{k,\lambda}$, we can rewrite the equations of motion in (S3) in a symmetric form

$$M_{nj}\ddot{\mathbf{R}}_{nj} = \mathbf{F}_{nj}^{(0)} + \mathbf{F}_{nj}^{\text{cav}}, \tag{S4a}$$

$$m_{k,\lambda}\ddot{\widetilde{\widetilde{q}}}_{k,\lambda} = -m_{k,\lambda}\omega_{k,\lambda}^2\widetilde{\widetilde{q}}_{k,\lambda} - \widetilde{\varepsilon}_{k,\lambda}\sum_{n=1}^{N_{\text{sub}}} d_{ng,\lambda}. \tag{S4b}$$

Here,

$$\mathbf{F}_{nj}^{\text{cav}} = -\sum_{k,\lambda}(\widetilde{\varepsilon}_{k,\lambda}\overset{\approx}{q}_{k,\lambda} + \frac{\widetilde{\varepsilon}_{k,\lambda}^2}{m_{k,\lambda}\omega_{k,\lambda}^2}\sum_{l=1}^{N_{\text{sub}}} d_{lg,\lambda})\frac{\partial d_{ng,\lambda}}{\partial \mathbf{R}_{nj}} \tag{S4c}$$

denotes the cavity force on each nucleus. Because we invoke periodic boundary conditions, it is logical to redefine

$$\widetilde{\varepsilon}_{k,\lambda} = \sqrt{N_{\text{cell}}}\varepsilon_{k,\lambda}, \tag{S4d}$$

to characterize the effective coupling between the cavity mode and each molecule in the periodic cell [where $\varepsilon_{k,\lambda}$, the true coupling strength between each cavity photon and individual

molecules, has been defined in Eq. (S2)]. Note that the equations of motion under periodic boundary conditions (Eq. (S4)) have a similar form as those without the periodic boundary conditions (Eq. (S3)). Not surprisingly, if we take $N_{\text{cell}} = 1$ (or if we assume $\widetilde{\varepsilon}_{k,\lambda} = \varepsilon_{k,\lambda}$), Eq. (S4) reduces to Eq. (S3).

b. Interpreting simulation results as Fabry–Pérot microcavities If CavMD is used to simulate VSC phenomena in Fabry–Pérot microcavities with N molecules and an experimental Rabi splitting Ω_N, since CavMD simulations use periodic boundary conditions with N_{sub} molecules in a periodic simulation cell, we can choose $\widetilde{\varepsilon}_{k,\lambda} \propto \sqrt{N_{\text{cell}}}$ so as to fit the experimentally observed Rabi splitting Ω_N.

Such a choice of $\widetilde{\varepsilon}_{k,\lambda}$, however, overestimates the light-matter coupling strength between the cavity modes and each individual molecule by a factor of $\sqrt{N_{\text{cell}}}$ relative to the true coupling strength $\varepsilon_{k,\lambda}$; see Eq. (S4d). Hence, when a VSC effect on molecular properties is obtained via CavMD, it is necessary to check the dependence on periodic boundary conditions (or the choice of the system size N_{sub}) to make sure that any observed VSC effect is not an artifact of the simulation.

In the main text, we have chosen to check the system size (or periodic boundary conditions) dependence as follows [2, 7]: (i) we keep the molecular density the same; (ii) when N_{sub} is enlarged by a factor of two, the effective light-matter coupling strength $\widetilde{\varepsilon}_{k,\lambda}$ is rescaled by a factor of $1/\sqrt{2}$ — such a balance between molecular number and coupling strength can maintain a fixed Rabi splitting; (iii) finally, by plotting the simulated VSC effect versus N_{sub}, the asymptotic results when N_{sub} approaches a macroscopic number should correspond to Fabry–Pérot microcavities.

This procedure for enlarging the system size has an experimental analog. As shown in Supplementary Figure 1, experimentally one can imagine enlarging the cavity length by a factor of two, which increases the cavity volume by a factor of two. Such an enlarging process can ensure both cavities have a cavity mode with the same frequency (red waves), although the light-matter coupling in the larger volume cavity is just $1/\sqrt{2}$ of the smaller one. When molecules with the same density are placed inside the cavities, the change of the light-matter coupling and the molecular number (which is proportional to the cavity volume) balance each other, yielding an unchanged Rabi splitting. This experimental approach for investigating large cavities matches our CavMD approach exactly.

c. Interpreting simulation results as plasmonic nanocavities Of course, CavMD can also be used to simulate plasmonic nanocavities under VSC where the molecular number

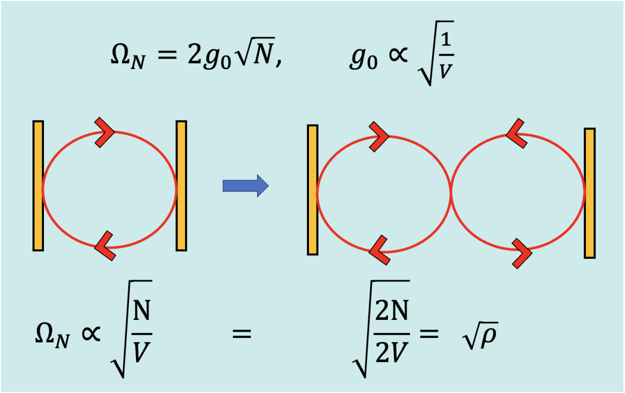

Supplementary Figure 1. Cartoon for the cavity size enlarging procedure. The Rabi splitting is the same when the cavity volume is increased by a factor of two and when molecules with the same density are filled in the cavity.

can be relatively small. Here, in CavMD we take $N_{\text{cell}} = 1$, so that $\widetilde{\varepsilon}_{k,\lambda} = \varepsilon_{k,\lambda}$ becomes the true coupling strength. Hence, for CavMD simulations with a given N_{sub}, one can interpret our CavMD dynamics as reporting on cavities with N_{sub} molecules form VSC. However, since plasmonic cavities usually have very large cavity loss, in order to obtain a convincing result for simulating plasmonic nanocavities, we need to add a very large cavity loss on top of CavMD.

d. Simulating external electric field driving When using CavMD to simulate driving nonequilibrium dynamics with an external electric field, we encounter a non-obvious choice as to what subsystem observable (internal to the cavity) we will choose to directly interact with the external electric field — e.g., will the external field drive directly the molecular vibrations or the electromagnetic cavity mode? In practice, we find that for nonequilibirum polariton dynamics, this choice of subsystem observable is immaterial: exciting either the (photonic or molecular) subsystem effectively pumps the polariton due to the fast coherent energy exchange between the two subsystems.

Mathematically, on top of the equations of motion in Eq. (S4), we can introduce the external driving on either the cavity modes or the molecules as follows. Assuming the interaction between the external field and the coupled cavity-molecular system as

$$V_{\text{ext}}(t) = -\boldsymbol{\mu}_S \cdot \mathbf{E}_{\text{ext}}(t) - \sum_{k,\lambda} \mu_c^{k,\lambda} \boldsymbol{\xi}_\lambda \cdot \mathbf{E}_{\text{ext}}(t) \tag{S5}$$

where $\boldsymbol{\mu}_S$ denotes the total dipole moment vector of the molecular system, $\mu_c^{k,\lambda} \equiv Q_c^{k,\lambda}\widetilde{q}_{k,\lambda}$ denotes the effective transition dipole moment (and $Q_c^{k,\lambda}$ denotes the effective charge) of the

cavity mode, and $\mathbf{E}_{\mathrm{ext}}(t)$ denotes the external time-dependent driving field, we can rewrite the equations of motion of the coupled cavity-molecular system as follows:

$$M_{nj}\ddot{\mathbf{R}}_{nj} = \mathbf{F}_{nj}^{(0)} + \mathbf{F}_{nj}^{\mathrm{cav}} + \mathbf{F}_{nj}^{\mathrm{ext}}(t), \quad \text{(S6a)}$$

$$m_{k,\lambda}\ddot{\widetilde{q}}_{k,\lambda} = -m_{k,\lambda}\omega_{k,\lambda}^2\widetilde{q}_{k,\lambda} - \widetilde{\varepsilon}_{k,\lambda}\sum_{n=1}^{N_{\mathrm{sub}}} d_{ng,\lambda} + \mathbf{F}_{k,\lambda}^{\mathrm{ext}}(t). \quad \text{(S6b)}$$

Here, the new added terms — the external force on each nucleus ($\mathbf{F}_{nj}^{\mathrm{ext}}(t)$) or cavity mode ($\mathbf{F}_{k,\lambda}^{\mathrm{ext}}(t)$) is defined as:

$$\mathbf{F}_{nj}^{\mathrm{ext}}(t) = Q_{nj}\mathbf{E}_{\mathrm{ext}}(t), \quad \text{(S7a)}$$

$$\mathbf{F}_{k,\lambda}^{\mathrm{ext}}(t) = Q_c^{k,\lambda}\mathbf{E}_{\mathrm{ext}}(t), \quad \text{(S7b)}$$

where Q_{nj} denotes the partial charge of each nucleus (if a classical force field is used to simulate the molecular subsystem).

In the main text, we have chosen to drive the molecular subsystem only (by setting $\mu_c = Q_c = 0$) because this choice avoids introducing an additional parameter Q_c, the value of which varies for different cavities and could hinder the universality of our finding. In Supplementary Figure 5, we will show that if the driving of the molecular subsystem is turned off and when the external field pumps only on the cavity modes (with a finite Q_c), a consistent simulation result is obtained as compared with case of pure molecular driving (where $Q_c = 0$; which is reported in the main text).

e. Simulation details We prepare 216 $^{12}CO_2$ molecules in a cubic cell with cell length 24.292 Å (45.905 a.u.) [8], which leads to the density of the liquid $^{12}CO_2$ system as 1.101 g/cm^3. We assume that a cavity formed by a pair of two parallel mirrors is placed along the z-axis. For a liquid $^{12}CO_2$ system under VSC, we consider only one cavity mode (but with two polarization directions x and y) at 2320 cm^{-1} (the peak frequency of ^{12}C=O asymmetric stretch), which is coupled to the molecular subsystem with an effective coupling strength $\widetilde{\varepsilon} = 2 \times 10^{-4}$ a.u. or 4×10^{-4} a.u.

Before pumping the system with an external laser pulse, we perform simulations to equilibrate the system. At 300 K, we first run the simulation for 150 ps to guarantee thermal equilibrium under a NVT (constant particle number, volume, and temperature) ensemble where a Langevin thermostat with a lifetime (i.e., inverse friction) of 100 fs is applied to the momenta of all particles (nuclei + photons). The resulting equilibrium configurations

are used as starting points for 40 consecutive NVE (constant particle number, volume, and energy) trajectories of length 20 ps. At the beginning of each trajectory the velocities are resampled by a Maxwell-Boltzmann distribution under 300 K. The intermolecular Coulombic interactions are calculated by an Ewald summation. The simulation step is set as 0.5 fs and we store the snapshots of trajectories every 2 fs.

We next perform nonequilibrium simulations under an external pulse. We start each simulation with an equilibrium geometry, which is chosen from the starting configurations of the above 40 NVE trajectories. At the beginning stage of each NVE trajectory, we apply an external pulse

$$\mathbf{E}_{\text{ext}}(t) = E_0 \cos\left(\omega t + \phi\right) \mathbf{e}_x \tag{S8}$$

to excite the molecular subsystem only. Here, the phase $\phi \in [0, 2\pi)$ is set as random. This pulse is turned on at $t_{\text{start}} = 0.1$ ps and is turned off at $t_{\text{end}} = 0.6$ ps. The incoming field is strong: $E_0 = 3.084 \times 10^7$ V/m (6×10^{-3} a.u.) and the fluence of the pulse is $F = 632$ mJ/cm^2. Note that in Supplementary Figure 5 we perform CavMD simulations by assuming that the incoming field is coupled to the cavity mode only; in Supplementary Figure 6, we also perform simulations when the incoming field is replaced by a Gaussian pulse.

Each nonequilibrium trajectory is run for 20 ps under a NVE ensemble and the physical properties are calculated by averaging over these 40 nonequilibrium trajectories. Note that the use of NVE trajectories when calculating equilibrium or nonequilibrium physical properties implies the assumption of zero cavity loss.

Apart from these NVE nonequilibrium simulations, in Supplementary Figure 4 below we also attach a Langevin thermostat with a friction lifetime τ_c to the photonic momenta to propagate the nonequilibrium simulations. Such a treatment can mimic the situation of a non-zero cavity loss, where the cavity lifetime is τ_c.

For the simulation of the liquid mixture of $^{13}CO_2$ and $^{12}CO_2$, we simply replace some ^{12}C atoms by the ^{13}C atoms and rerun the above equilibrium + nonequilibrium simulations.

As stated in the manuscript and above, we refer to our cavity system as "a single cavity mode" because the photonic energies for the two polarization directions are the same, and only a single photon peak appears in the IR spectroscopy — so there is only a single mode *from a spectroscopic point of view.* Of course, we could also refer to our cavity system as a system with "two energy-degenerated cavity photons with different polarization directions", but this description seems a bit lengthy.

f. Definition of the CO_2 force field The CO_2 force field used in the manuscript [2] largely resembles Ref. [9] except for the use of an anharmonic C=O bond potential instead of a harmonic one. According to Ref. [9], in the harmonic limit, this force field can capture macroscopic thermal equilibrium observables and also the equilibrium IR spectroscopy of liquid CO_2 that are consistent with experiments.

In this CO_2 force field, the intramolecular interaction is characterized by

$$V_{\mathrm{g}}^{(n)} = V_{\mathrm{CO}}(R_{n1}) + V_{\mathrm{CO}}(R_{n2}) + \frac{1}{2}k_\theta\left(\theta_n - \theta_{\mathrm{eq}}\right)^2. \quad \text{(S9)}$$

Here, R_{n1} and R_{n2} denote the lengths of two C=O bonds,and θ_n and θ_{eq} denote the O−C−O angle and the equilibrium angle. The form of V_{CO} is defined as follows:

$$V_{\mathrm{CO}}(r) = D_r\left[\alpha_r^2\Delta r^2 - \alpha_r^3\Delta r^3 + \frac{7}{12}\alpha_r^4\Delta r^4\right], \quad \text{(S10)}$$

where $\Delta r = r - r_{\mathrm{eq}}$. Eq. (S10) is a fourth-order Taylor expansion of a Morse potential $V_{\mathrm{M}}(r) = D_r[1 - \exp(-\alpha_r\Delta r)]^2$. The parameters are taken as follows: $r_{\mathrm{eq}} = 1.162$ Å (2.196 a.u.), $D_r = 127.13$ kcal mol^{-1} (0.2026 a.u.), and $\alpha_r = 2.819$ Å^{-1} (1.492 a.u.) are chosen to fit the harmonic potential used in Ref. [9] in the harmonic limit and the value of D_r takes the bond dissociation energy of O=CO at room temperature [10]. The other parameters in Eq. (S9) are given as $k_\theta = 108.0$ kJ mol^{-1}rad^{-2} (0.0861 a.u.), and $\theta_{\mathrm{eq}} = 180$ deg.

The pairwise intermolecular potential is characterized by the Lennard–Jones potential ($V_{\mathrm{LJ}}^{(nl)}$) plus the Coulombic interactions between atoms ($V_{\mathrm{Coul}}^{(nl)}$):

$$V_{\mathrm{inter}}^{(nl)} = V_{\mathrm{LJ}}^{(nl)} + V_{\mathrm{Coul}}^{(nl)}, \quad \text{(S11)}$$

where the superscript n, l denote the indices of different molecules. The form of $V_{\mathrm{LJ}}^{(nl)}$ is

$$V_{\mathrm{LJ}}^{(nl)} = \sum_{i\in n}\sum_{j\in l} 4\epsilon_{ij}\left[\left(\frac{\sigma_{ij}}{R_{ij}}\right)^{12} - \left(\frac{\sigma_{ij}}{R_{ij}}\right)^{6}\right], \quad \text{(S12)}$$

where R_{ij} ($i \in n$ and $j \in l$) denotes the distance between atoms in molecules n and l. For parameters, $\epsilon_{\mathrm{CC}} = 0.0559$ kcal mol^{-1} (8.9126×10^{-5} a.u.), $\sigma_{\mathrm{CC}} = 2.800$ Å (5.291 a.u.), $\epsilon_{\mathrm{OO}} = 0.1597$ kcal mol^{-1} (2.5454×10^{-4} a.u.), $\sigma_{\mathrm{OO}} = 3.028$ Å (5.722 a.u.), $\epsilon_{\mathrm{CO}} = \sqrt{\epsilon_{\mathrm{CC}}\epsilon_{\mathrm{OO}}}$, and $\sigma_{\mathrm{CO}} = (\sigma_{\mathrm{CC}} + \sigma_{\mathrm{OO}})/2$. The form of $V_{\mathrm{Coul}}^{(nl)}$ is

$$V_{\mathrm{Coul}}^{(nl)} = \sum_{i\in n}\sum_{j\in l}\frac{Q_iQ_j}{4\pi\epsilon_0 R_{ij}}. \quad \text{(S13)}$$

For parameters, $Q_{\mathrm{C}} = 0.6512$ $|e|$, and $Q_{\mathrm{O}} = -0.3256$ $|e|$ (where e denotes the charge of the electron).

Given the CO_2 force field defined above, one can easily calculate the cavity-free force $\mathbf{F}_{nj}^{(0)}$ (see Eq. (S6)) as a function of the nuclear configurations by standard molecular dynamics packages like LAMMPS [11]. In CavMD, at each time step, we also need to evaluate the molecular dipole moment projected along direction $\boldsymbol{\xi}_\lambda = \mathbf{e}_x, \mathbf{e}_y$:

$$d_{ng,\lambda} = \left[Q_{\mathrm{O}}\left(\mathbf{R}_{n\mathrm{O}_1} + \mathbf{R}_{n\mathrm{O}_2}\right) + Q_{\mathrm{C}}\mathbf{R}_{n\mathrm{C}}\right] \cdot \boldsymbol{\xi}_\lambda \tag{S14}$$

and its derivative $\partial d_{ng,\lambda}/\partial \mathbf{R}_{nj}$, which is just the partial charge of each nucleus (Q_{O} or Q_{C}). For the calculation of the IR spectrum (see below), the total dipole moment $\boldsymbol{\mu}_S$ projected along direction $\boldsymbol{\xi}_\lambda$ is calculated by $\boldsymbol{\mu}_S \cdot \boldsymbol{\xi}_\lambda = \sum_{n=1}^{N_{\mathrm{sub}}} d_{ng,\lambda}$.

g. Spectroscopy of polaritons and dark modes In the main text, the global infrared (IR) absorption spectrum is calculated by Fourier transforming the dipole auto-correlation function from equilibrium NVE trajectories [12–15]:

$$n(\omega)\alpha(\omega) = \frac{\pi\beta\omega^2}{2\epsilon_0 V c}\frac{1}{2\pi}\int_{-\infty}^{+\infty} dt\ e^{-i\omega t}\left\langle \sum_{i=x,y} \left(\boldsymbol{\mu}_S(0)\cdot\mathbf{e}_i\right)\left(\boldsymbol{\mu}_S(t)\cdot\mathbf{e}_i\right)\right\rangle . \tag{S15}$$

Here, $\alpha(\omega)$ denotes the absorption coefficient, $n(\omega)$ denotes the refractive index, V is the volume of the system (i.e., the simulation cell), $\mathbf{e}_i$ denotes the unit vector along direction $i = x, y$, and $\boldsymbol{\mu}_S(t)$ denotes the *total* dipole moment of the molecules at time t, where $\boldsymbol{\mu}_S(t) = \sum_{nj} Q_{nj}\mathbf{R}_{nj}(t)$. See the above definitions around Eq. (S13) for the values of the partial charges Q_{nj} of each nucleus (the true nuclear charge for nucleus nj plus the electronic shielding effect), which is assumed to be a constant within the classical force field. In Eq. (S15), the summation over x and y is a summation over the two possible polarizations of the relevant cavity mode (where the cavity is assumed to be oriented along the z-direction; see Fig. 1 in the main text for the cavity setup).

When evaluating the nonequilibirum local IR spectroscopy for each molecule after the external pulse pumping, given a nonequilibrium NVE trajectory, we calculate

$$n(\omega)\alpha_{\mathrm{local},l}(\omega) = \frac{\pi\beta\omega^2}{2\epsilon_0 V c}\frac{1}{2\pi}\int_{-\infty}^{+\infty} dt\ e^{-i\omega t}\left\langle \boldsymbol{\mu}_l(0)\cdot\boldsymbol{\mu}_l(t)\right\rangle . \tag{S16}$$

Here, $\boldsymbol{\mu}_l(t) = \sum_j Q_{lj}\mathbf{R}_{lj}(t)$ denotes the dipole moment for molecule l.

Note that Eq. (S15) describes the collective behavior of the molecular dipole system, which reflects the polaritonic response under VSC. By contrast, Eq. (S16) provides information about the dynamics of each individual molecule, which is composed mostly of vibrational dark modes (since the molecular number in the simulation, N_{sub} is always 216 or more and the polaritonic contribution of each molecule is small).

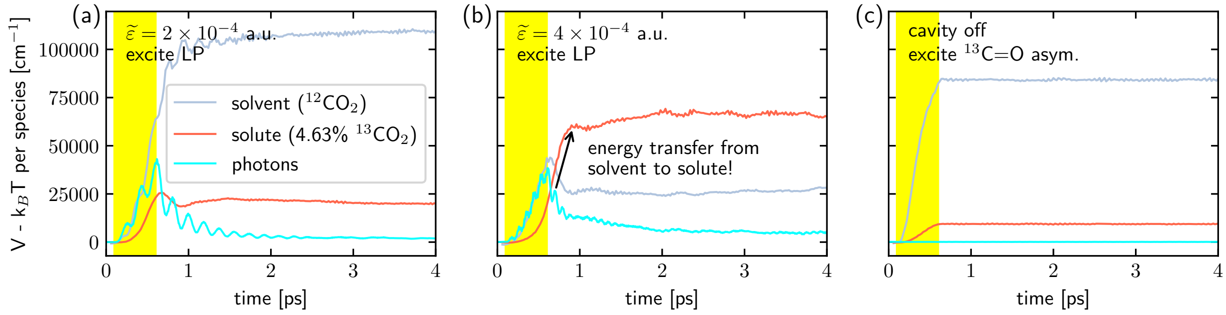

Supplementary Figure 2. Replot of the data in Fig. 3 of the manuscript except here the dynamics of the C=O bond energy per species (not per molecule) is shown. In other words, we plot the total amount of energy in the $^{12}CO_2$ and $^{13}CO_2$ species. The photonic potential energy is also plotted as the cyan line. As shown in Fig. (b), first the solvent (blue line) gains energy from interacting with the external field (i.e., during the yellow window); subsequently, after a small delay, the solvent transfers a large amount of energy to the solute (red line) after the field is turned off (i.e. after the yellow window). As the solvent polariton transfers energy to the solute molecules or solvent dark modes, the photonic energy is also reduced. This data shows unambiguously show that there is an ultrafast (sub-ps) vibrational energy transfer from the solvent to the solute molecules. Outside the cavity (Fig. (c)), such a fast intermolecular vibrational energy transfer is impossible, highlighting the benefits of forming vibrational polaritons and the use of a nonlinear mechanism to promote intermolecular vibrational energy transfer. To reduce signal fluctuations, we have applied a time-series moving average to the curves with neighboring four data points.

SUPPLEMENTARY NOTE 1 | ULTRAFAST INTERMOLECULAR ENERGY TRANSFER FROM SOLVENT TO SOLUTE

In Supplementary Figure 2, we replot Fig. 3 in the manuscript but here we show the C=O bond energy per molecular species (not per molecule). In Supplementary Figure 2b, it is very clear that there is an ultrafast vibrational energy transfer from the solvent to the solute species, which is absent either outside the cavity (as in Supplementary Figure 2c) or when the solvent LP does not have a suitable frequency to support the nonlinear absorption of the solute molecules (as in Supplementary Figure 2a).

The timescale of intermolecular vibrational energy transfer in Supplementary Figure 2b is short which might lead the reviewer to question our interpretation of the dynamics above. In order to demonstrate the time-resolved energy transfer dynamics even more clearly, in

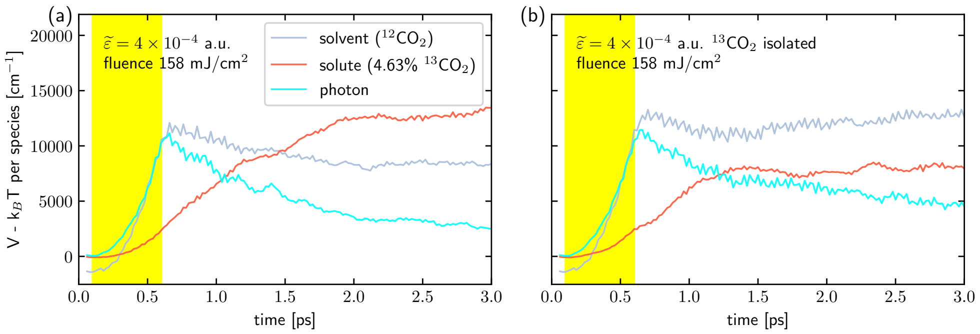

Supplementary Figure 3. (a) Similar plot as Supplementary Figure 2b except that the pulse fluence is reduced from 632 mJ/cm^2 to 158 mJ/cm^2. Under a smaller pulse fluence, after the pulse excitation (the yellow window), the energy transfer from the $^{12}CO_2$ solvent to the $^{13}CO_2$ solute species can be identified more easily. (b) Replot of Fig. (a) except that the intermolecular interactions involving $^{13}CO_2$ molecules are turned off. In the absence of the $^{13}CO_2$ intermolecular interactions, the $^{12}CO_2$ $\rightarrow$ $^{13}CO_2$ intermolecular vibrational energy transfer is greatly suppressed.

Supplementary Figure 3 we perform two additional simulations and plot the potential energy *per species*.

For the first simulation (to be compared against the simulation condition in Fig. 3b of the manuscript (or Supplementary Figure 2b)), in Supplementary Figure 3a we have reduced the pulse fluence from $F = 632$ mJ/cm^2 to $F = 158$ mJ/cm^2. Such a reduction of the pulse fluence can increase the polariton lifetime and give better time-resolved energy transfer dynamics than that in Supplementary Figure 2b. In Supplementary Figure 3a, after the pulse excitation (yellow window), we find that both the solvent and photonic energies are transferred to the solute molecules, showing that forming solvent polaritons can indeed facilitate intermolecular vibrational energy transfer. Although the solute concentration is only 4.63%, most the input energy is accumulated in the solute molecules at $t = 3$ ps.

For the second simulation, we further remove the intermolecular interactions involving the $^{13}CO_2$ species. In other words, Supplementary Figure 3b is an *unphysical* simulation where the intermolecular interactions between each $^{13}CO_2$ and other molecules ($^{12}CO_2$ or $^{13}CO_2$) are artificially turned off. Although Supplementary Figure 3b is unphysical, this plot shows unambiguously that, as compared with Supplementary Figure 3a, the energy transfer from the $^{12}CO_2$ species to the $^{13}CO_2$ species is greatly suppressed, demonstrating the importance of intermolecular interactions in facilitating energy transfer. Overall, Supplementary Figure 3b shows that our mechanism of energy accumulation in the solute species after the solvent

polariton pumping is indeed a many-body mechanism and intermolecular interactions play an important role.

SUPPLEMENTARY NOTE 2 | EFFECT OF CAVITY LOSS

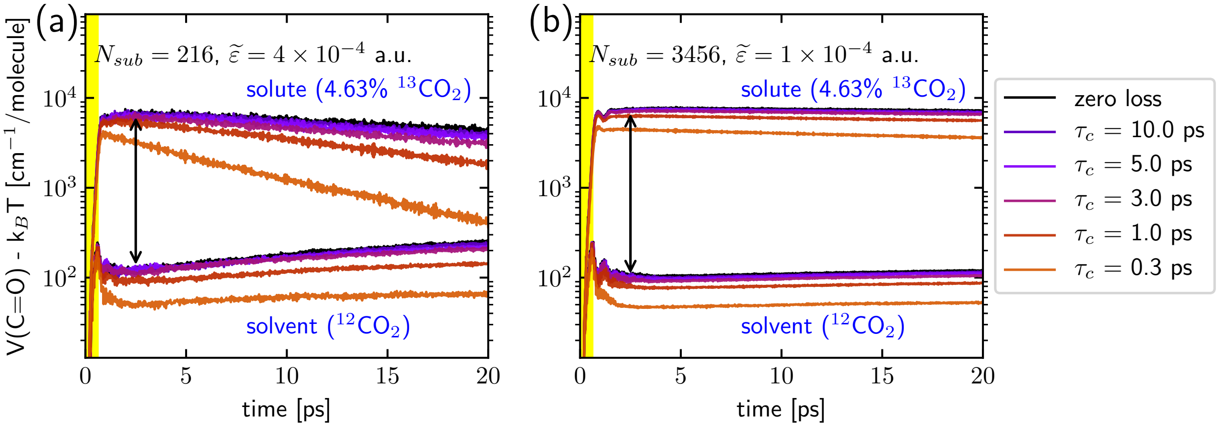

Supplementary Figure 4. (a) C=O bond energy dynamics per $^{13}CO_2$ solute and $^{12}CO_2$ solvent molecules when the molecular system size is $N_{\mathrm{sub}} = 216$ and $\widetilde{\varepsilon} = 4\times10^{-4}$ a.u. (the same simulation parameters as Fig. 3b in the main text). Black to orange lines denote different cavity losses, ranging from zero loss to a cavity lifetime of 10.0, 5.0, 3.0, 1.0, and 0.3 ps. (b) The corresponding dynamics when the system size is enlarged by 16 times and the Rabi splitting is fixed (by taking $\widetilde{\varepsilon} = 1\times10^{-4}$ a.u.). These simulations indicate that, for Fabry–Pérot cavities (which corresponds to the limit when N_{sub} is large), cavity loss does not meaningfully alter the reported dynamics in Fig. 3b once the cavity lifetime is larger than 1 ps.

The reported results in the main text did not include cavity loss. Here, in Supplementary Figure 4a, we rerun the main finding (Fig. 3b) in the main text by adding a cavity loss on top of the original simulation. All other parameters and simulation details are the same as in Fig. 3b. Color lines from black to orange denote the choice of different cavity loss (from zero cavity loss to a cavity lifetime of 10.0, 5.0, 3.0, 1.0, and 0.3 ps). The top lines represent the C=O bond energy dynamics per $^{13}CO_2$ solute molecule, while the bottom lines represent that of $^{12}CO_2$ solvent molecules. As shown in Supplementary Figure 4a, the simulation results are robust against the cavity loss when the cavity lifetime is longer (or the same as) 3 ps.

As discussed in Supplementary Methods, when using CavMD to simulate Fabry–Perot

microcavities, we need to enlarge the system size to check the convergence of the simulation results. Supplementary Figure 4b plots the corresponding C=O bond energy dynamics when the molecular number in the simulation cell is enlarged from $N_{\text{sub}} = 216$ to 3456 (by 16 times). Accordingly, in order to maintain the fixed Rabi splitting, the effectively light-matter coupling strength is reduced from $\widetilde{\varepsilon} = 4 \times 10^{-4}$ a.u. to $\widetilde{\varepsilon} = 1 \times 10^{-4}$ a.u. In this relatively large molecular system, cavity loss plays a role only when the cavity lifetime is smaller than 1 ps.

We can understand the cavity loss dependence of the dynamics as follows. The C=O bond dynamics can be divided into two phases: the polariton relaxation ($t < 1$ ps; since the polariton lifetime is 0.6 ps; see Fig. 2h in the main text) and later-time vibrational relaxation and energy transfer after the polariton decay ($t > 1$ ps). The former dynamics are dominated by the polariton, and the later dynamics are dominated by the dark modes. As far as the first phase (polariton relaxation) is considered, when the cavity loss is very large (or when the cavity lifetime is smaller than 1 ps), because cavity loss becomes an important channel for the energy dissipation, less polariton energy can be transferred (or dephased) to the vibrational dark modes, leading to a significantly altered C=O bond energy dynamics. By contrast, when the cavity lifetime becomes larger than 1 ps, the early-time dynamics is not altered by the cavity loss (for Supplementary Figure 4a,b). In later times (the second phase), for the dynamics of each individual molecule, since the polariton contribution is just $1/N_{\text{sub}}$ and the dark-mode dynamics dominate the molecular response, cavity loss no longer plays a meaningful role when N_{sub} is large (Supplementary Figure 4b). For Supplementary Figure 4a, since N_{sub} is not very large ($N_{\text{sub}} = 216$), cavity loss plays a more important role for the later-time dynamics than that in Supplementary Figure 4b ($N_{\text{sub}} = 3456$), with meaningful differences when the cavity lifetime is smaller than 3 ps.

Overall, when CavMD is used to simulate Fabry–Pérot microcavities, the system size enlarging process in Supplementary Figure 4 shows that cavity loss cannot meaningfully alter the reported dynamics when the cavity lifetime is greater than 1 ps (which is the usual parameter in Fabry–Perot cavities). Of course, Supplementary Figure 4 also indicates that, for small-volume cavities (such as plasmonic nanocavities) under VSC, a large cavity loss may suppress the selectivity of energy transfer.

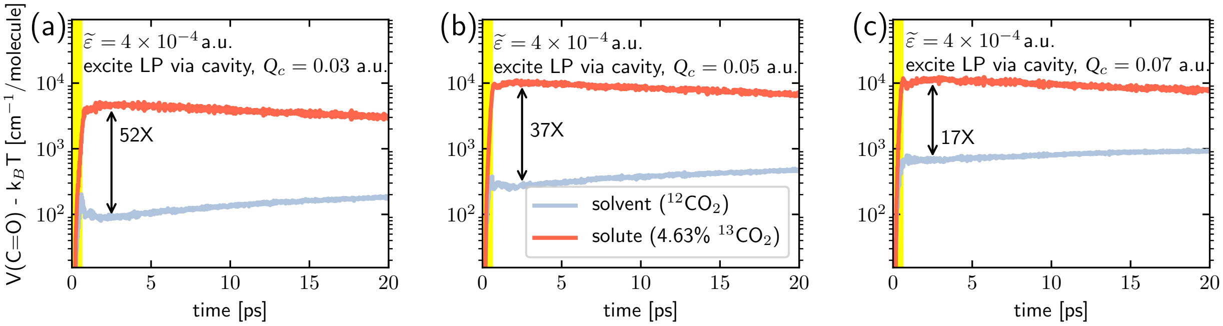

Supplementary Figure 5. Replot of Fig. 3b except for the change that the incoming field interacts only with the cavity mode via an effective charge Q_c (rather than interacting with the molecular subsystem). All the other simulation details are the same as in Supplementary Figure 5b. In (a-c), we plot the C=O bond dynamics when Q_c takes the values 0.03, 0.05, and 0.07 a.u., respectively. Note that similar amount of energy is infused to the coupled cavity-molecular system in both Fig. 3b (of the main text) and Fig. a (here).

SUPPLEMENTARY NOTE 3 | EFFECT OF PUMPING CAVITY MODES

The reported results in the main text have assumed that the incoming field interacts with the molecular subsystem only. In Supplementary Figure 5, we replot Fig. 3b (a key figure in the main text) by assuming that the incoming field interacts with the cavity mode only via the following interaction form $-Q_c\widetilde{\widetilde{q}}_c\mathbf{E}_{\mathrm{ext}}(t)$, where we have defined Q_c as the effective charge of the cavity mode in Supplementary Methods. Keeping all the other simulation details are the same as in Fig. 3b, Supplementary Figure 5 plots the C=O bond dynamics for the solute and solvent molecules for different choices of Q_c: (from left to right) $Q_c =$ 0.03, 0.05, and 0.07 a.u. We have found that when $Q_c = 0.03$ a.u., Supplementary Figure 5a largely reproduces Fig. 3b, showing that exciting either the molecular or the cavity mode yields similar results when a similar amount of the input energy is infused to the coupled cavity-molecular system. Admittedly, for these simulations there is an ambiguity insofar as the fact that we must choose a value for Q_c; nevertheless, our conclusions are robust provided that Q_c is chosen so that we dump the same amount of energy into the cavity in Supplementary Figure 5 as in Fig. 3b of the main text.

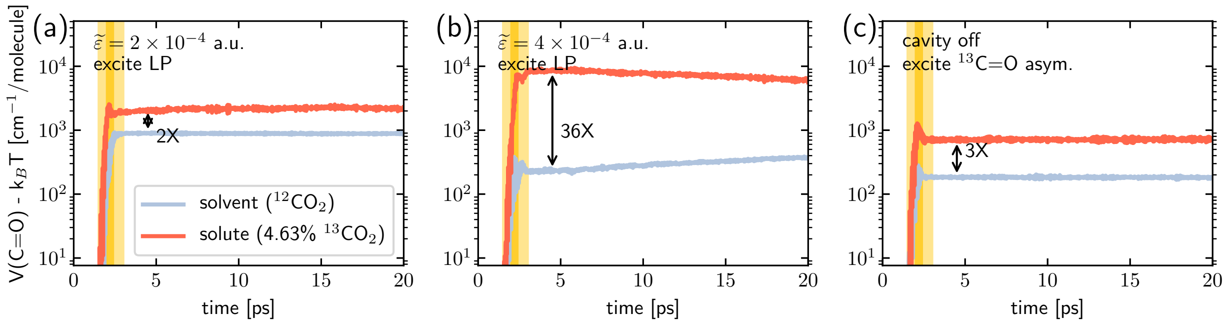

Supplementary Figure 6. Replot of Fig. 3 in the main text except for the use of an Gaussian pulse defined in Eq. (S17) instead of a continuous wave pulse (see Eq. (S8)). See text below for details.

SUPPLEMENTARY NOTE 4 | EFFECT OF A GAUSSIAN PULSE

The reported results in the main text have assumed a pulse in the form of Eq. (S8). Here, we replace this pulse form by a Gaussian pulse:

$$\mathbf{E}_{\rm ext}(t) = E_0 \exp\left[-2\ln(2)\frac{(t - t_0 - 4\tau_{\rm FWHM})^2}{\tau_{\rm FWHM}^2}\right]\cos(\omega t)\mathbf{e}_x, \tag{S17}$$

where we set $E_0 = 3.084 \times 10^7$ V/m (6×10^{-3} a.u.), $\tau_{\rm FWHM} = 0.5$ ps, $t_0 = 0.01$ ps. In Supplementary Figure 6, we replot Fig. 3 in the main text for the case that we excite with this Gaussian pulse; all the other parameters are the same as in Fig. 3. The yellow vertical region denotes the time window when the molecular system experiences the peak of the Gaussian pulse. The results are largely the same as in Fig. 3. Note that, since the parameters for the Gaussian pulse have been chosen to resemble the wave pulse in Eq. (S8), a similar amount of energy is pumped into the coupled cavity-molecular system as in Fig. 3 of the main text.

SUPPLEMENTARY NOTE 5 | EFFECT OF THE INPUT PULSE FLUENCE

Supplementary Figure 7 plots the dependence of selectivity (i.e., the C=O bond energy ratio between the $^{13}CO_2$ and $^{12}CO_2$ molecules at $t = 2.5$ ps) as a function of the incoming pulse fluence when the simulation parameters are identical to that in Fig. 3b except for the pulse fluence. The transient selectivity goes through a maximum as a function of the pulse fluence, confirming our assertion that energy transfer to the $^{13}CO_2$ species is dominated by a nonlinear transition: at low intensities this mechanism is inefficient, while at high intensities the $^{13}CO_2$ species energy absorption becomes saturated.

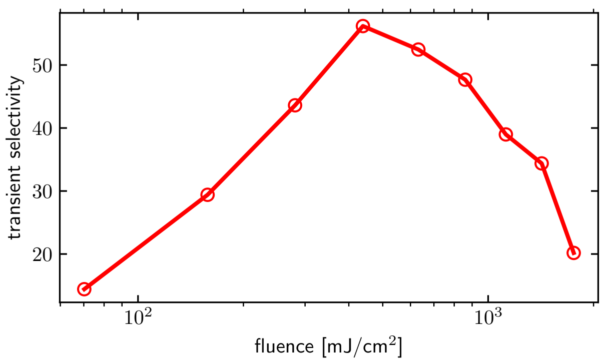

Supplementary Figure 7. C=O bond energy ratio between the average $^{13}CO_2$ and the average $^{12}CO_2$ molecule at $t = 2.5$ ps (i.e., the transient selectivity labeled in Fig. 3b) as a function of the input pulse fluence. Except for the change of pulse fluence, all the other parameters are the same as in Fig. 3b of the main text. Note that the transient selectivity goes through a maximum as a function of the pulse fluence. This inversion behavior comes from two competing effects: on the one hand, increasing the pulse fluence can enhance the nonlinear absorption for the $^{13}CO_2$ molecules which tends to increase the selectivity; one the other hand, enhancing the pulse fluence over a threshold saturates the energy absorption of all the molecules, which tends to reduce the selectivity.

SUPPLEMENTARY NOTE 6 | EFFECT OF WEAK OR STRONG COUPLING

In Supplementary Figure 2 and Supplementary Figure 3, we have shown the uniqueness of our finding — under a suitable solvent polariton frequency, it is possible to achieve an ultrafast (sub-ps) and strong intermolecular vibrational energy transfer from the $^{12}CO_2$ solvent to the $^{13}CO_2$ solute molecules after pumping the solvent polariton. Here, we are interested in another aspect of our finding: under which conditions can we highly excite the few $^{13}CO_2$ solute molecules, especially do we need strong light-matter interactions?

It is clear that if the external field interacts only with the molecular bright mode rather than with the cavity mode, since forming a solvent polariton increases the effective dipole moment of the system, the presence of strong coupling will lead to more total absorption than one would find for the case of a few isolated $^{13}CO_2$ molecules in a cavity (with weak coupling). Then, if the energies of the solvent and solute allow for the nonlinear $0 \to 2$ nonlinear mechanism as described in the main body of the text, we might expect more energy accumulation in the $^{13}CO_2$ molecules (when dynamics proceed through a polariton

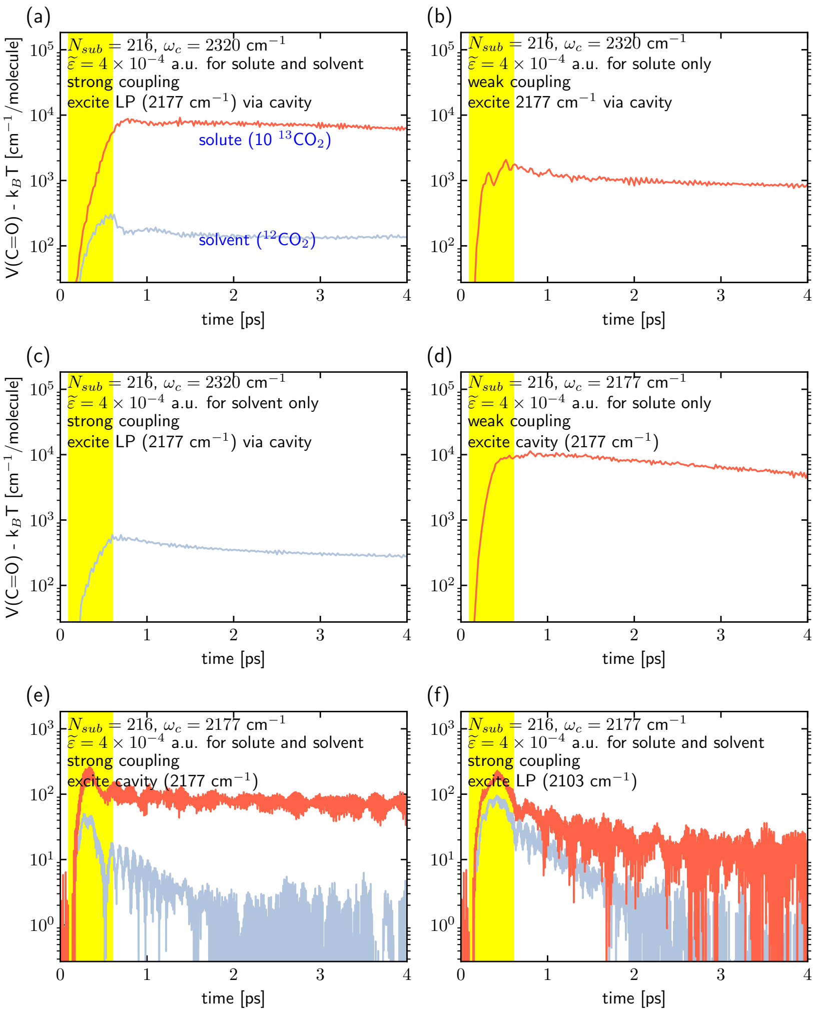

Supplementary Figure 8. C=O bond energy dynamics per $^{13}CO_2$ solute (red) or $^{12}CO_2$ solvent (gray) molecule when an external pulse peaked at 2177 cm^{-1} excites the cavity mode (not molecules) with $Q_c = 0.05$ a.u. (a) Similar plot as Fig. 3b in the manuscript when the cavity mode (ω_c = 2320 cm^{-1}) forms strong coupling with the solute/solvent system. (b) Cavity (ω_c = 2320 cm^{-1}) contains the $^{13}CO_2$ species only. Other details are the same as in Fig. (a) (including the pulse excitation at 2177 cm^{-1}). Here, the solute is not strongly pumped because, without solvent, the incoming excitation is off-resonant. (c) Cavity (ω_c = 2320 cm^{-1}) contains the $^{12}CO_2$ host only and the other details are the same as Fig. (a) (including the pulse excitation at the LP frequency 2177 cm^{-1}). The solvent energy absorption is larger than Fig. (a) because there is no solute molecules for energy accumulation. (d) Cavity (ω_c = 2177 cm^{-1}) contains the $^{13}CO_2$ species only. The pulse excites at 2177 cm^{-1} (the cavity mode frequency here). From Fig. (d), we find that, if we could turn off solvent-cavity interactions, we could simply build a cavity whose frequency is close the $^{13}CO_2$ frequency, pump at that same frequency, and excite the solute to a highly excited state without strong coupling. (e) Cavity (ω_c = 2177 cm^{-1}) coupled to both the solute and solvent molecules and the pulse excites at 2177 cm^{-1} (the cavity mode frequency here). (f) Similar plot as in Fig. (e) except that the external pulse frequency is set to 2103 cm^{-1} (the LP frequency for this case). For Fig. (e), the solute is not strongly excited since the pumping is off-resonant because the presence of light-matter coupling $\widetilde{\varepsilon}$ shifts the energy of the cavity mode. For Fig (f), even when pumping the LP, we still find no enhancement of the $^{13}CO_2$ because there is no nonlinear mechanism (and energy match) to promote two quanta of excitation on a solute molecule. In Figs. (a)-(f), the cavity loss lifetime is set to τ_c = 0.4 ps (= 83 cm^{-1}); this choice of τ_c is large enough such that, if the solvent were to interact with the cavity, there would be strong coupling. However, if only the solute interacts with the cavity, there is weak coupling — the Rabi splitting is less than the width of the cavity mode (as relevant to Figs. (b) and (d)) Altogether, in the presence of many $^{12}CO_2$ solvent molecules, there is no means of exciting the $^{13}CO_2$ molecules strongly and preferentially without illuminating the $^{12}CO_2$ solvent polariton and activating the nonlinear ($0 \rightarrow 2$) mechanism described in the main body of the text.

formed by strong coupling with the solvent molecule) than we would with simple excitation of a few $^{13}CO_2$ molecules (where there is no strong coupling at all). Indeed, this is what we have found.

One might wonder if the conclusions above are limited by the assumption that light interacts with the molecules directly (and not through the cavity). To that extent, we will now consider the parameter limit when only the cavity mode (not the molecules) interacts with the external pulse. We are interested in the situations when the few $^{13}CO_2$ molecules can be highly excited (no matter weak or strong coupling). In order to make a fair comparison, we have taken the light-matter coupling between the cavity mode and each individual molecule to be the same ($\widetilde{\varepsilon} = 4 \times 10^{-4}$ a.u.) throughout this comparison, meaning that the cavity volume is taken the same (i.e., we are interested in the same type of cavity setup).

We are interested in six different cases, which have been described in detail in the caption of Supplementary Figure 8. Supplementary Figure 8d shows that the $^{13}CO_2$ absorption can be enhanced in a suitably chosen cavity (ω_c = 2177 cm^{-1}) under weak coupling if only a few $^{13}CO_2$ molecules are inside the cavity (i.e., we have manually removed the $^{12}CO_2$ solvent molecules). However, if we restrict ourselves to a molecular system with a large amount of $^{12}CO_2$ solvent molecules plus a few $^{13}CO_2$ solute molecules, Supplementary Figure 8e,f show even when the cavity frequency is tuned to ω_c = 2177 cm^{-1}, because the presence of the solvent molecules will non-resonantly form a LP with the cavity mode (with ω_{LP} = 2103 cm^{-1}), neither exciting the cavity mode nor exciting the LP frequency can lead to a strong excitation of the $^{13}CO_2$ molecules. Ultimately, as also shown in Supplementary Figure 3, to achieve a strong *selective* excitation of the $^{13}CO_2$ solute molecules in the presence of a bath fo $^{12}CO_2$ solvent molecules, the best approach is indeed to match the solvent mode with the cavity mode and excite the LP frequency — in other words, the approach prescribed in the main body of the present paper.

SUPPLEMENTARY NOTE 7 | COMBINED EFFECTS OF CAVITY LOSS, PUMPING CAVITY MODES, AND A GAUSSIAN PULSE

Lastly, let us consider the situation when an external Gaussian pulse pumps a lossy cavity mode directly. Such a parameter setting is similar to the input-output theory of optical cavities [16]. In this framework, the quantum coupling between an external electric

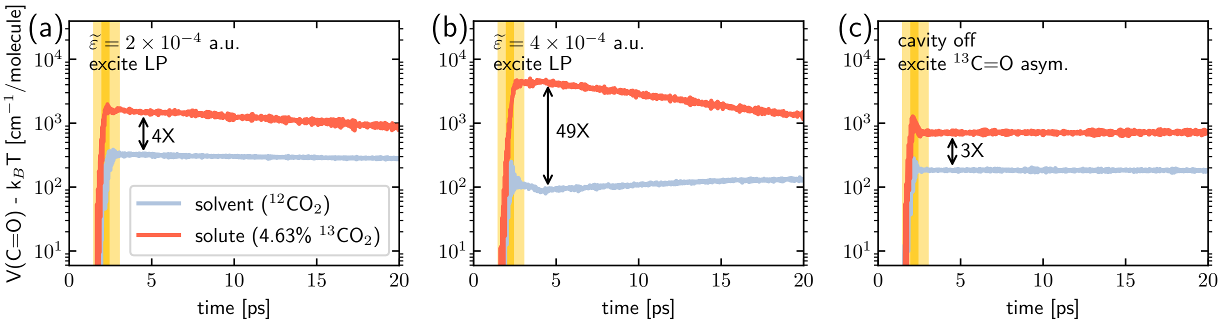

Supplementary Figure 9. Replot of Fig. 3 in the main text with the following differences: (i) an Gaussian pulse defined in Eq. (S17) is used instead of the wave pulse in Eq. (S8); (ii) in Figs. (a) and (b), the external field excites the cavity mode only with $Q_c = 0.028$ a.u. and the cavity lifetime is set as $\tau_c = 0.75$ ps. Again, we find large selective excitation of the solute molecules.

field and the cavity modes in a planar optical cavity can be defined as

$$\hat{H}_{\rm pump} = i\hbar \int \frac{d^2\mathbf{k}_\parallel}{(2\pi)^2} \sum_\sigma \left[\eta_\sigma^{\rm fr}(\mathbf{k}_\parallel) \tilde{E}_\sigma^{\rm inc}(\mathbf{k}_\parallel, t) \hat{a}_{c,\sigma}^\dagger(\mathbf{k}_\parallel) - \eta_\sigma^{\rm fr*} \tilde{E}_\sigma^{\rm inc*}(\mathbf{k}_\parallel, t) \hat{a}_{c,\sigma}(\mathbf{k}_\parallel) \right]. \quad \text{(S18)}$$

Here, $\hat{a}_{c,\sigma}^\dagger(\mathbf{k}_\parallel)$ and $\hat{a}_{c,\sigma}(\mathbf{k}_\parallel)$ denote the creation and annihilation operators of cavity photon with an in-plane wave vector $\mathbf{k}_\parallel$ and polarization direction σ, $\tilde{E}_\sigma^{\rm inc}(\mathbf{k}_\parallel, t)$ denotes the Fourier transform of the incident field $\tilde{E}_\sigma^{\rm inc}(\mathbf{r}, t)$, and $\eta_\sigma^{\rm fr}(\mathbf{k}_\parallel)$ is the coefficient proportional to the transmission amplitude of the front mirrors of the planar optical cavity when the incident light has an in-plane wave vector $\mathbf{k}_\parallel$. The finite transmittivity of the front and back mirrors of the cavity leads to the transmitted and reflected electric fields, which take the following form:

$$\hat{E}_\sigma^{\rm tr}(\mathbf{k}_\parallel, t) = \kappa_\sigma^{\rm back}(\mathbf{k}_\parallel) \hat{a}_{c,\sigma}(\mathbf{k}_\parallel, t), \quad \text{(S19a)}$$

$$\hat{E}_\sigma^{\rm refl}(\mathbf{k}_\parallel, t) = E_\sigma^{\rm inc}(\mathbf{k}_\parallel, t) + \kappa_\sigma^{\rm fr}(\mathbf{k}_\parallel) \hat{a}_{c,\sigma}(\mathbf{k}_\parallel, t), \quad \text{(S19b)}$$

where $\kappa_\sigma^{\rm fr,back}(\mathbf{k}_\parallel)$ denotes the coefficient proportional to the transmission amplitude of the front or back mirrors. The cavity loss can be treated as a Lindblad form on top of the density matrix of the cavity field $\hat{\rho}$:

$$\mathcal{L}[\hat{\rho}] = \int \frac{d^2\mathbf{k}_\parallel}{(2\pi)^2} \frac{\gamma_\sigma^{\rm rad}(\mathbf{k}_\parallel)}{2} \left[2\hat{a}_{c,\sigma}(\mathbf{k}_\parallel) \hat{\rho} \hat{a}_{c,\sigma}^\dagger(\mathbf{k}_\parallel) - \hat{a}_{c,\sigma}(\mathbf{k}_\parallel) \hat{a}_{c,\sigma}^\dagger(\mathbf{k}_\parallel) \hat{\rho} - \hat{\rho} \hat{a}_{c,\sigma}(\mathbf{k}_\parallel) \hat{a}_{c,\sigma}^\dagger(\mathbf{k}_\parallel) \right], \quad \text{(S20)}$$

where $\gamma_\sigma^{\rm rad}(\mathbf{k}_\parallel)$ denotes the radiative decay rate of the cavity. In the follows the nonradiative decay rate of the cavity will be disregarded.

Under the simplest case when $\eta_\sigma^{\mathrm{fr}}(\mathbf{k}_\parallel) = \eta_\sigma^{\mathrm{back}}(\mathbf{k}_\parallel)$, $\kappa_\sigma^{\mathrm{fr}}(\mathbf{k}_\parallel) = \kappa_\sigma^{\mathrm{back}}(\mathbf{k}_\parallel)$, $\gamma_\sigma^{\mathrm{rad}}(\mathbf{k}_\parallel) = -2\eta_\sigma(\mathbf{k}_\parallel)\kappa_\sigma(\mathbf{k}_\parallel)$, and also assuming normal incidence $\mathbf{k}_\parallel = 0$, given the transmittivity of the cavity mirrors as $T \ll 1$, we have

$$\eta = \frac{cT}{2l_z}\sqrt{\frac{l_z}{\pi\hbar\omega_c}}, \tag{S21a}$$

$$\kappa = -T\sqrt{\frac{\pi\hbar\omega_c}{l_z}}, \tag{S21b}$$

$$\gamma = \frac{cT^2}{l_z}. \tag{S21c}$$

Let us connect the parameters in input-output theory to those (i.e., τ_c and Q_c) in CavMD. For a cavity mode of frequency $\omega_c = 2320$ cm^{-1}, the corresponding cavity length is $l_z = 2.16$ μm (with fundamental quantum number). Assuming $T = 0.025$, we compute the cavity lifetime as

$$\tau_c = \frac{1}{\gamma} = \frac{l_z}{cT^2} = 0.75 \text{ ps}. \tag{S22}$$

We further connect η to Q_c. Using $\hat{a}_c^\dagger - \hat{a}_c = \sqrt{2\omega_c/\hbar}\hat{q}_c$ and the classical coupling as $-Q_c\widetilde{\widetilde{q}}_c\mathbf{E}_{\mathrm{ext}}(t)$ in CavMD, we may write

$$Q_c = \hbar\eta\sqrt{\frac{2\omega_c}{\hbar}} = \frac{cT}{\sqrt{2\pi l_z}} = 0.028 \text{ a.u.} \tag{S23}$$

Given the above values ($\tau_c = 0.75$ ps and $Q_c = 0.028$ a.u.), and also taking the Gaussian pulse defined above, when the other parameters are kept the same as Fig. 3 in the main text, we rerun CavMD simulations and plot Supplementary Figure 9 (in a similar way as Fig. 3 in the main text). Again, a large selectivity is observed inside the cavity.

SUPPLEMENTARY NOTE 8 | A QUANTUM ILLUSTRATION OF OUR FINDING

The numerical findings reported in the main text can be justified by a quantum picture. As shown in the left box of Supplementary Figure 10, for an anharmonic potential surface with three energy levels $|0\rangle$ (the black rectangle), $|1\rangle$ (the red rectangle), and $|2\rangle$ (the yellow rectangle), when a strong pulse at the frequency which is half of the $|0\rangle \to |2\rangle$ transition pumps the molecular system, multiphoton nonlinear absorption can occur, which is facilitated by a virtual state (i.e., a second-order process in a Fermi's golden rule calculation).

Beyond this simple textbook picture of multiphoton nonlinear absorption, polaritons can also enhance the nonlinear absorption of molecules (see the middle box of Supplementary

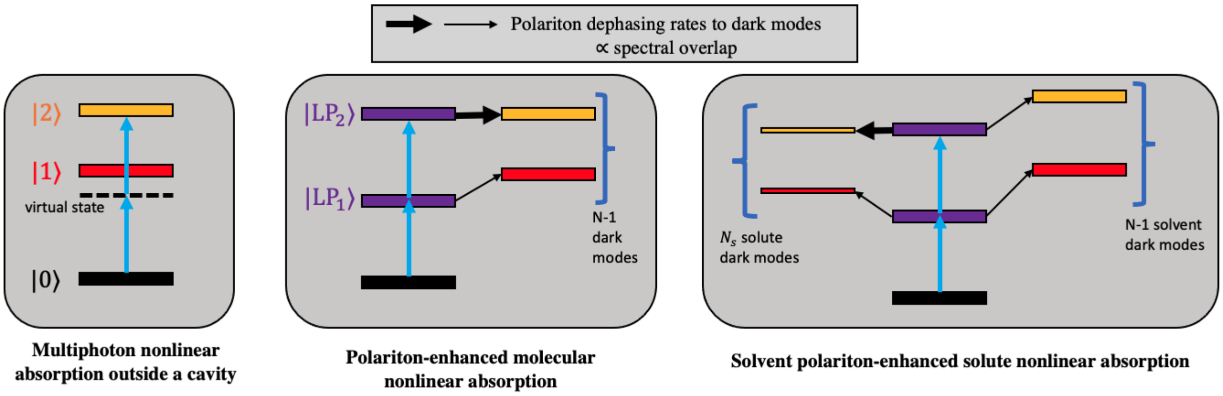

Supplementary Figure 10. A quantum illustration of selective exciting the solute molecules by pumping the solvent polariton. (left box) Illustration of multiphoton nonlinear absorption outside a cavity; (middle box) illustration of polariton-enhanced molecular nonlinear absorption; (right box) illustration of our finding in the main text, i.e., when the two LP state $|\mathrm{LP}_2\rangle$ is near resonance with the second excited states of the solute dark modes, strongly pumping the LP may lead to a dominate dephasing channel from the LP to the second excited state of a set of solute molecules. The black arrows denote the polaritonic dephasing channels to the dark modes and the thickness of the arrows represents the rate of the dephasing rate (due to spectral overlap).

Figure 10). Considering the case when N identical anharmonic molecules are coupled to a cavity mode and strong coupling is formed, the first excited state of the LP ($|\mathrm{LP}_1\rangle$, the purple rectangle) can dephase to the $N-1$ first excited states of the dark modes ($|1_d\rangle$). Similarly, the second excited state of the LP ($|\mathrm{LP}_2\rangle$, the purple rectangle) can dephase to the second excited states of the dark modes ($|2_d\rangle$). A Fermi's golden rule calculation shows that the polaritonic dephasing rate is proportional to the spectral overlap between the initial (polaritonic) and the final (dark) states. Now, when $|\mathrm{LP}_2\rangle$ is near resonance with $|2_d\rangle$, the large spectral overlap between the $|\mathrm{LP}_2\rangle$ and $|2_d\rangle$ states will lead to a very fast dephasing process for the $|\mathrm{LP}_2\rangle$ state. When the incoming field is strong enough, $|\mathrm{LP}_2\rangle$ can be meaningfully populated and outperform the dephasing of the $|\mathrm{LP}_1\rangle$ state, which leads to polariton-enhanced molecular nonlinear absorption.

Finally, as shown in the right box of Supplementary Figure 10, we consider the case when N solvent molecules form strong coupling with a cavity mode and $|\mathrm{LP}_2\rangle$ has a very small spectral overlap with the solvent $|2_d\rangle$ states. In this case, polariton-enhanced molecular nonlinear absorption is greatly suppressed for the solvent molecules. Now, if we add a small

concentration of the solute molecules to the system, the presence of the solute molecules will not alter the frequencies of the polaritons. If the $|2_d\rangle$ states of the solute molecules (the thin yellow rectangle) are near resonance with the $|\mathrm{LP}_2\rangle$, a strong excitation of the LP will result in enhanced nonlinear absorption for the solute molecules only.

In principle, one can construct master equations to describe the above mechanism. However, from our perspective, such a master equation would depend critically on the four dephasing rates ($|\mathrm{LP}_1\rangle$ or $|\mathrm{LP}_2\rangle$ to the solute or solvent degrees of freedom) and the reliability of any such master equation would be questionable without *ab initio* modeling (ideally quantum modeling) — which is currently not practical. In this sense, quantum master equations may not be more realistic than our fully atomistic classical CavMD simulations. For this reason, we have chosen not to propagate the master equations. Nevertheless, the quantum illustration in Supplementary Figure 10 should help understand our findings from a quantum perspective.

REFERENCES

[S1] T. E. Li, J. E. Subotnik, and A. Nitzan, Cavity molecular dynamics simulations of liquid water under vibrational ultrastrong coupling, Proc. Natl. Acad. Sci. **117**, 18324 (2020).

[S2] T. E. Li, A. Nitzan, and J. E. Subotnik, Cavity molecular dynamics simulations of vibrational polariton-enhanced molecular nonlinear absorption, J. Chem. Phys. **154**, 094124 (2021).

[S3] C. Cohen-Tannoudji, J. Dupont-Roc, and G. Grynberg, *Photons and Atoms: Introduction to Quantum Electrodynamics* (Wiley, New York, 1997) pp. 280–295.

[S4] T. E. Li, A. Nitzan, and J. E. Subotnik, On the origin of ground-state vacuum-field catalysis: Equilibrium consideration, J. Chem. Phys. **152**, 234107 (2020).

[S5] T. S. Haugland, E. Ronca, E. F. Kjønstad, A. Rubio, and H. Koch, Coupled Cluster Theory for Molecular Polaritons: Changing Ground and Excited States, Phys. Rev. X **10**, 041043 (2020).

[S6] J. Flick, M. Ruggenthaler, H. Appel, and A. Rubio, Atoms and Molecules in Cavities, from Weak to Strong Coupling in Quantum-Electrodynamics (QED) Chemistry, Proc. Natl. Acad. Sci. **114**, 3026 (2017).

[S7] T. E. Li, A. Nitzan, and J. E. Subotnik, Collective vibrational strong coupling effects on molecular vibrational relaxation and energy transfer: Numerical insights via cavity molecular

dynamics simulations, Angew. Chemie Int. Ed. , anie.202103920 (2021).

[S8] L. Martínez, R. Andrade, E. G. Birgin, and J. M. Martínez, PACKMOL: A package for building initial configurations for molecular dynamics simulations, J. Comput. Chem. **30**, 2157 (2009).

[S9] R. T. Cygan, V. N. Romanov, and E. M. Myshakin, Molecular Simulation of Carbon Dioxide Capture by Montmorillonite Using an Accurate and Flexible Force Field, J. Phys. Chem. C **116**, 13079 (2012).

[S10] B. d. Darwent, *Bond dissociation energies in simple molecules* (U.S. National Bureau of Standards, 1970).

[S11] S. Plimpton, Fast Parallel Algorithms for Short-Range Molecular Dynamics, J. Comput. Phys. **117**, 1 (1995).

[S12] D. A. McQuarrie, *Statistical Mechanics* (Harper-Collins Publish- ers, New York, 1976).

[S13] M.-P. Gaigeot and M. Sprik, Ab Initio Molecular Dynamics Computation of the Infrared Spectrum of Aqueous Uracil, J. Phys. Chem. B **107**, 10344 (2003).

[S14] S. Habershon, G. S. Fanourgakis, and D. E. Manolopoulos, Comparison of path integral molecular dynamics methods for the infrared absorption spectrum of liquid water, J. Chem. Phys. **129**, 074501 (2008).

[S15] A. Nitzan, *Chemical Dynamics in Condensed Phases: Relaxation, Transfer and Reactions in Condensed Molecular Systems* (Oxford University Press, New York, 2006).

[S16] I. Carusotto and C. Ciuti, Quantum fluids of light, Rev. Mod. Phys. **85**, 299 (2013).
